# Connecting past and present

**DOI:** 10.7554/eLife.101212

**Published:** 2024-08-13

**Authors:** Sihan Yang, Anastasia Kiyonaga

**Affiliations:** 1 University of California, San Diego San Diego United States

**Keywords:** working memory, serial dependence, neural representation, multivariate analysis, magnetoencephalography, Human

## Abstract

A neural signature of serial dependence has been found, which mirrors the attractive bias of visual information seen in behavioral experiments.

**Related research article** Fischer C, Kaiser J, Bledowski C. 2024. A direct neural signature of serial dependence in working memory. *eLife* **13**:RP99478. doi: 10.7554/eLife.99478.

When looking at a scene, we frequently blink, make eye and head movements, and have our vision blocked by moving external objects. Yet, instead of changing abruptly in response to these events, our visual experience remains fluid and continuous.

Research suggests that previously seen stimuli influence what we perceive in the present. A phenomenon, called serial dependence, indicates that the visual system anticipates that an object seen now is the same as the one seen a moment ago (Fischer and Whitney, 2014). This could make new objects look more similar to recently viewed stimuli than they are. For example, a green object may be perceived as teal by someone who has recently seen a similar blue object.

This so-called "attractive bias" applies to many features, from color and orientation to aesthetic value and facial expression – but only when successive stimuli are reasonably similar (Manassi et al., 2023). Rather than taking a rolling average of recent moments, it is thought that past and current visual inputs may be weighted in the brain and selectively integrated to strengthen relevant signals (Cicchini et al., 2024). This could explain why our visual experience seems continuous.

Scientists have been disputing the true ecological purpose of serial dependence and when it emerges in the processing stream. Determining the neural functions that give rise to the behavior would illuminate whether serial dependence actually changes what we see, or influences our memories and decisions that guide actions. Now, in eLife, Cora Fischer, Jochen Kaiser and Christoph Bledowski at Goethe University Frankfurt report having identified a neural signature that tracks the hallmark serial dependence effect (Fischer et al., 2024).

Recent research has detected neural traces of previous stimuli that may linger to induce attractive bias (Barbosa et al., 2020; Ranieri et al., 2022). Others have found that while behavioral reports showed bias towards previous stimuli, response patterns in the visual cortex showed bias away from the previous stimuli instead (Hajonides et al., 2023; Sheehan and Serences, 2022). This hints that a later, post-perceptual process must override this repulsion, but it had not been identified. Fischer et al. found evidence for later neural representations that are attractively shifted toward previous features, matching the attractive behavioral effect.

The team used whole-brain magnetoencephalography (MEG) and quantitative modelling to study the neural responses of humans completing a series of behavioral tasks. Participants underwent multiple trials in which they were presented with two consecutive stimuli (dots moving in one direction and different colored dots moving in another direction) and asked to remember the dot directions. After a short pause, they were cued to recall the direction of one of the dot colors. Memory of the cued stimulus showed typical serial dependence, where the reported directions were shifted a few degrees in the direction of the dots cued for recall in the previous trial (Figure 1).

**Figure 1.**
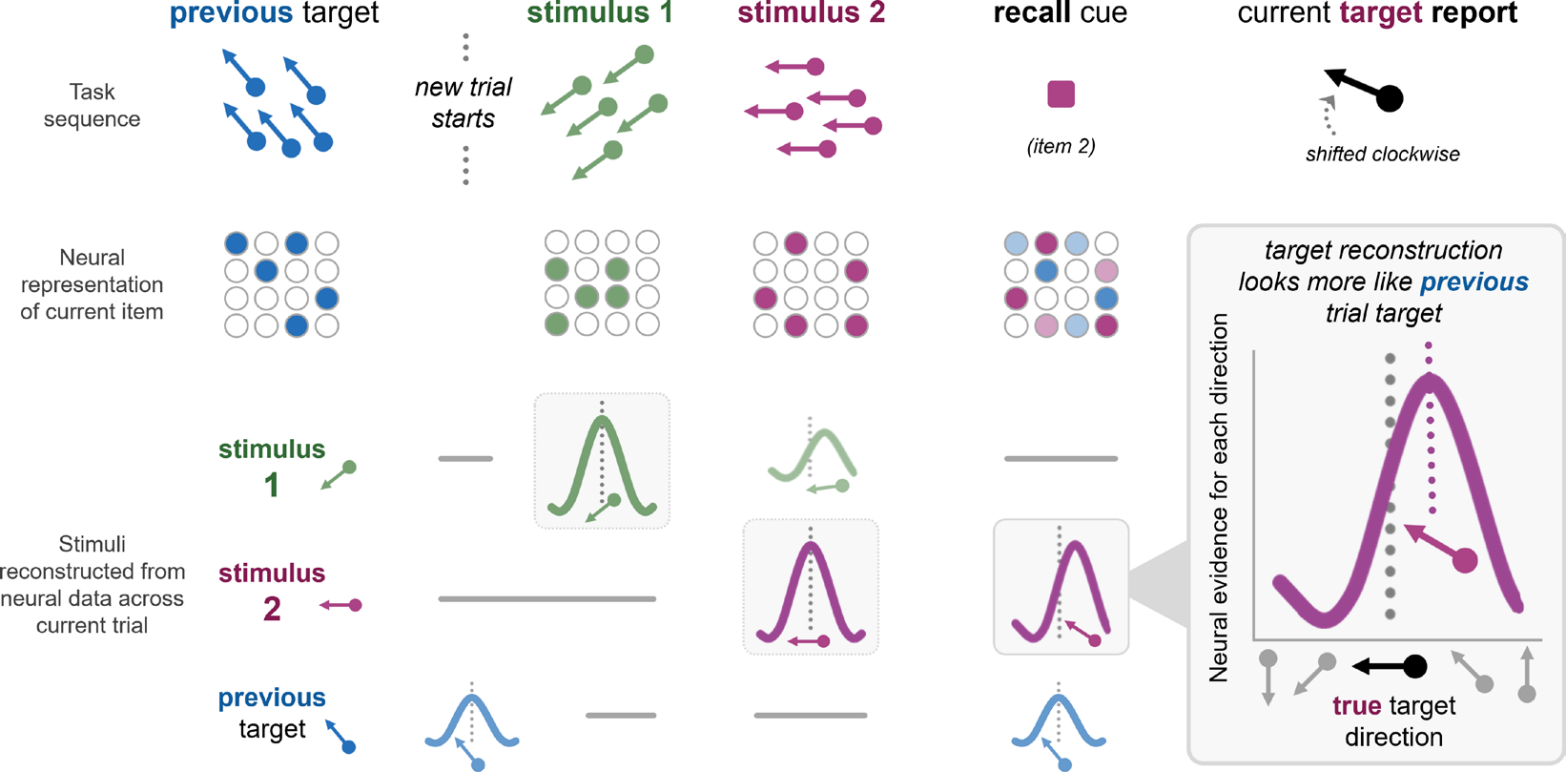
Neural representations of current stimuli are biased by previously relevant content. Participants completed a visual working memory task consisting of multiple trials (top). Each trial consisted of two sequential arrays of moving dot stimuli of different colors and motion directions (stimulus 1 and 2; represented as green and purple). Participants were asked to remember the direction of motion of each of the arrays. After a brief pause, a colored recall cue (purple) signaled participants to recall the direction of motion of the corresponding colored dots. Behavioral reports for this target stimulus (right) were biased toward the target direction of the recalled stimulus from the previous trial (blue; left), consistent with serial dependence. The second row shows schematics of the measured neural representations triggered by each dot stimulus below the corresponding stimulus, focusing only on the stimulus representation that was most relevant at each major task phase (also corresponding to the highlighted quantitative model reconstructions shown below). The neural response to the recall cue was influenced by the information for the previous trial target (right; represented as a combination of blue and purple dots). Schematic reconstructions of neural activity across each phase of the trial (bottom) display a peak that reflects the feature value (i.e., motion direction) with the greatest neural evidence. The current trial target reconstruction peaks at a value that is biased in the motion direction of the previous trial target. The reconstruction shows no bias when stimulus 2 (purple) is initially seen. When asked to recall the motion direction, the peak moves to reflect bias in the motion direction of the previous trial target (blue).

Fischer et al. then applied an inverted encoding model to the MEG data to measure representations of both the current remembered motion directions and the motion direction from the previous trial (Sprague et al., 2018). This analysis reconstructed an estimate of the stimulus information in the pattern of neural signals as a proxy for the underlying representation: a reconstruction with a peak centered around the true feature value would reflect an unbiased representation, but one shifted off-center would suggest a representation bias one way or another.

They found that motion representations appeared unbiased during encoding (i.e., perception). However, target representations became attractively biased during memory recall, when neural activity shifted toward the target direction from the previous trial. This would suggest participants were remembering a different direction than shown, i.e., they were remembering a direction that was more similar to the previous trial.

The findings of Fischer et al. are unique in capturing a neural bias that emerges at later, post-encoding time points and support the idea that serial dependence lags behind the initial perception. The timing could signify a decision-making phenomenon or indicate that memory representations become noisier and subject to bias over time. Neural representations of the motion direction from the previous trial target also appeared reactivated at the start of both a new trial and the cue for recall in the new trial, and representations of the first stimulus in a trial became attractively biased at the second stimulus onset – hinting that transition points between perceptual episodes may spur (or unveil) the integration process (Sols et al., 2017). However, these MEG signals were not specific to one brain region, so the critical circuits and computations warrant further investigation.

Serial dependence has captivated the vision science community and triggered burgeoning research into how this occasional bug imparts adaptive function. Now, neuroscience is catching up to reveal the physiology that supports serial dependence and our effortless perceptual experience. The work of Fischer et al. paves the way to probe how the visual system determines what information to assimilate, how it manifests at different levels of processing, and how it interacts with other features of the context and natural world.
